# Case report: Post-streptococcal pustulosis after subcutaneous injection of secukinumab

**DOI:** 10.3389/fmed.2023.1217545

**Published:** 2023-06-29

**Authors:** Xiaoqing Lang, Hongmei Bi, Yingjie Zhang, Hongzhou Cui, Hongye Liu, Ling Ren, Yao Dang, Shuping Guo

**Affiliations:** Department of Dermatology, First Hospital of Shanxi Medical University, Taiyuan, China

**Keywords:** secukinumab, group A β-hemolytic streptococcus, pustulosisacuta generalisata, skin rash, case report

## Abstract

An acute diffuse pustular eruption occurred in a patient after secukinumab injection and then the clinical presentation has been related to streptococcus infection after it has been isolated from throat swabs. Pustulosisacuta generalisata was definitively diagnosed. Antibiotic treatment had a poor effect, but the response to glucocorticoids was better.

## Introduction

Pustulosisacuta generalisata (PAG), also known as post-streptococcal pustulosis (PSP), is a rare sterile pustulosis. PAG is characterized by a scattered symmetric eruption of sterile pustules with an inflammatory halo arising on normal skin and predominantly affecting the hands and feet, and to a lesser extent the arms, legs, and trunk. The pustules may be confluent. PAG is linked to a group A β-hemolytic streptococcus infection. Complications of PAG include mucosal involvement, glomerulonephritis, and arthralgias. The differential diagnosis for PAG includes acute generalized exanthematous pustulosis (AGEP) and generalized pustular psoriasis (GPP). Establishing a PAG diagnosis may be difficult. The pustular eruption spontaneously resolves without relapses or use of external drugs; however, a prolonged and severe course should be treated systemically. Herein, we report the case of a 55-year-old woman with typical clinical and histologic findings of PAG emerging after a pharyngeal infection. There was no personal or family history of skin diseases.

## Case reports

**Figure 1:**
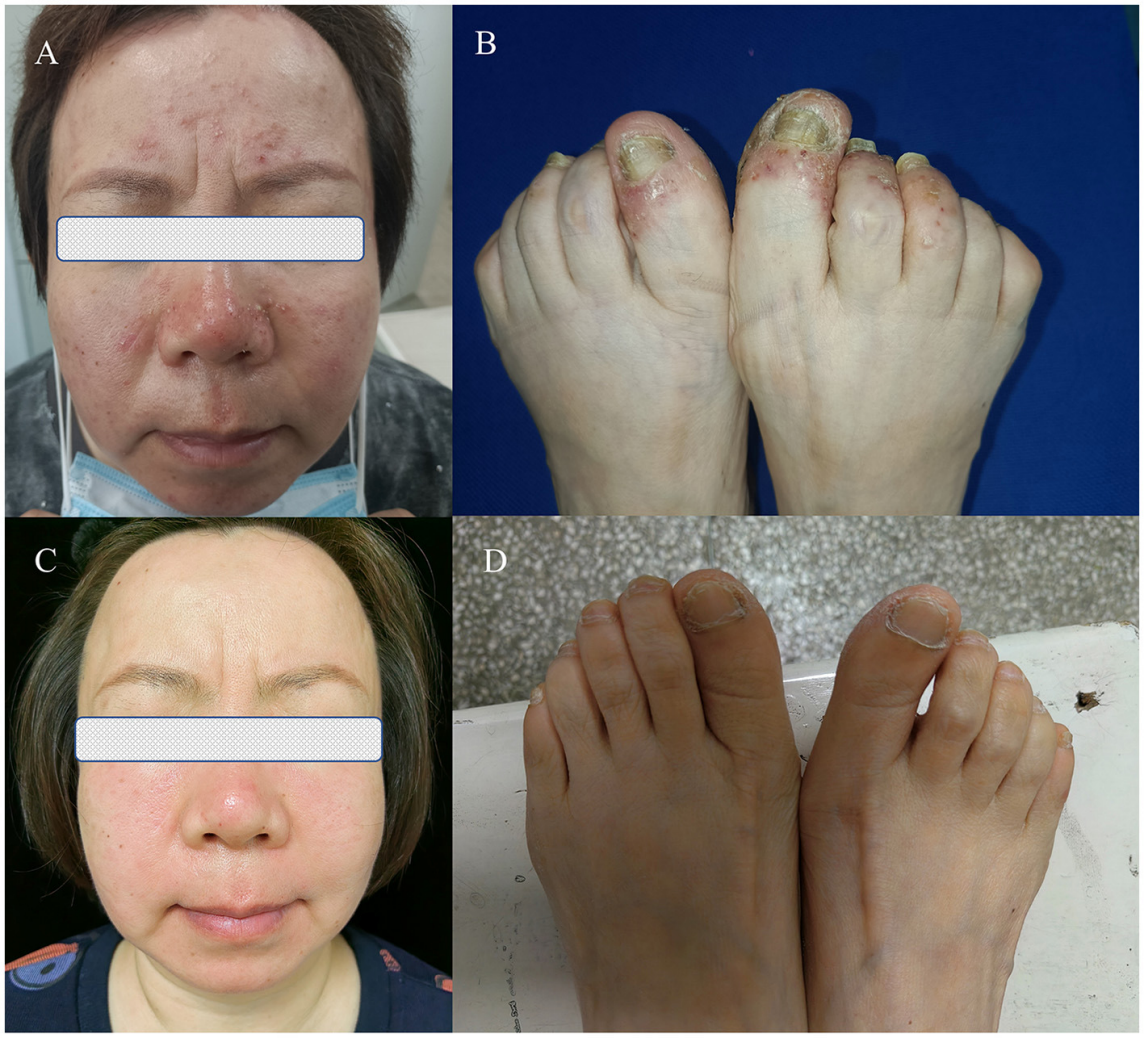
**(A, B)** Pre-treatment photograph. **(C, D)** Post-treatment photograph.

A 55-year-old woman was admitted to First Hospital of Shanxi Medical University our hospital with a pustular skin disease that had developed over the previous 15 days. She reported erythema and plaques on the palms of the hands and soles of the feet 3 days after receiving the first dose of an inactivated coronavirus disease 2019 (COVID-19) vaccine in April 2021 (Changchun Institute of Biological Products Co., Ltd., Changchun, China). In May 2022, red plaques of different sizes appeared on the scalp and limbs with a thick layer of white scales on the surface. The fingernails and toenails had a gray-white, unclean color with erythema around the nails. The pathologic evaluation suggested psoriasis. Three days after the second subcutaneous injection of secukinumab in the local hospital (150 mg/week), the patient presented with a series of symptoms, including throat erythema, tongue pain, dysphagia, mandibular lymphadenopathy, and fever (up to 39.4°C). There were pustules and erosions on the oral mucosa. In addition, both external auditory canals were swollen, tender, and painful with a yellow exudate that was treated with cefuroxime sodium and metronidazole. The antibiotics had no apparent effect; in fact, erythema and pustules arose on day 10 of antibiotic treatment (Figure 1).

The pustules were distributed symmetrically and were most numerous on the trunk and face and, to a lesser extent, on the hands and feet (Figure 1). The physical examination was otherwise normal.

**Figure 2:**
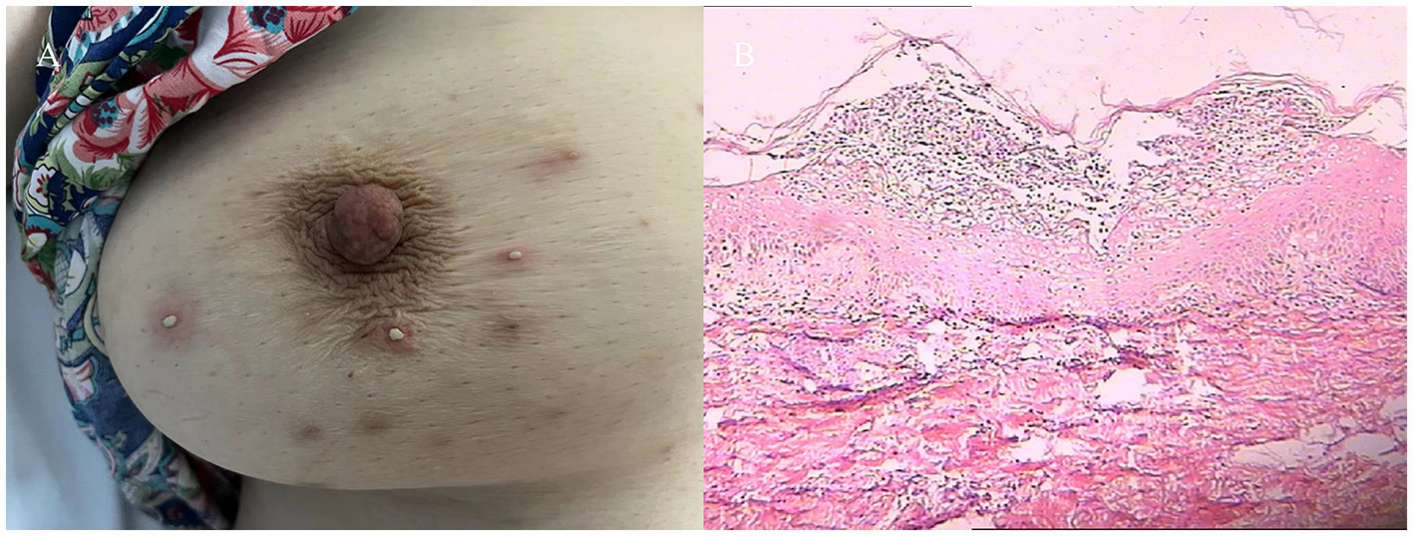
**(A)** Pustules on the right side of the chest. **(B)** Histopathology on the right side of the chest.

The white blood cell count on admission was 8.1 × 10^9^/L (reference range, 3.5–5 × 10^9^/L), the absolute value of neutrophils was 8.5 × 10^9^/L (reference range, 1.8–6.3 × 10^9^/L), and the percentage of neutrophils was 81.7% (reference range, 40–75%). The erythrocyte sedimentation rate 1 day after hospital admission was 110 mm (reference range, 0–20 mm) in the first hour. The high-sensitivity C-reactive protein level was 119.04 mg/L (reference range, 0–6 mg/L). Cutaneous swabs from the pustules were negative for bacteria, viruses, and fungi. Group A β-hemolytic streptococcus was isolated from throat swabs. Because of the results from the throat swabs, the anti-streptolysin titer was determined (300 IU/mL; reference range, 0–200 IU/mL). Blood cultures obtained on admission remained negative after 1 week of incubation. The patient was HLA-A2- and HLA-B35-negative. A chest x-ray showed no relevant pathologic findings. A urinalysis was within normal limits. The histologic evaluation of a biopsy specimen from the skin on the right side of the chest revealed a sub-corneal pustule and a perivascular lymphocytic infiltrate in the upper dermis (Figure 2). Based on the history and clinical features, we made a diagnosis of PAG following a cutaneous infection by group A β-hemolytic streptococcus.

The patient was initially treated with levofloxacin hydrochloride injection (0.4 g once daily for 6 days), and azithromycin injection (0.5 g once daily for 6 days), but no significant improvement was observed. methylprednisolone sodium succinate injection (40 mg once daily for 8 days)was administered, the erythema and pustules completely disappeared, and the presenting symptoms resolved (Figure 2). The hematologic indices returned to normal ranges. Subsequently, ixekizumab injection was used on a regular basis to treat psoriasis. There were no reported adverse effects of the medication and no recurrences after 1 year of follow-up evaluations.

## Discussion

Several cutaneous complications have been reported with COVID-19 vaccines (1). Herein we describe a patient with the onset of psoriasis shortly after COVID-19 vaccination. According to a systematic review, COVID-19 vaccines can lead to the new onset and/or exacerbations of psoriasis (2). Biological agents have been shown to be a positive and effective means to treat psoriasis. Indeed, several studies have shown that biological agents may aggravate or induce infection (3). Our patient presented with a sore throat, oral erosions, and otitis externa with an acute diffuse pustular eruption due to group A β-hemolytic streptococcus that developed 2 days after receiving the second injection of secukinumab. In agreement with the case report by Eren et al. (4), our patient was definitively diagnosed with PAG.

PAG is a post-infectious disease in individuals who are susceptible to streptococcal infections, and who are genetically prone to develop pustular skin disease (5). Only 25 patients with PAG have been described worldwide (4). Patrizi et al. (6) reported a 6-year-old boy with a diffuse acute pustular eruption that appeared after an episode of erysipelas. It has been suggested that PAG occurs after pharyngeal infection with group A β-hemolytic streptococcus. Our patient had clinical findings consistent with the characteristics of the case reported by Patrizi et al. (6). Further studies are necessary to completely understand the pathogenesis underlying PAG, from which several hypotheses have been postulated. Indeed, deposition of immune complexes formed by streptococcal antigens and antibodies (type III immune reaction) may be involve (7).

Antibodies cross-reactive with the immunodominant epitope of group A streptococcal carbohydrate and cardiac valves, skin, kidneys, and other tissues have been demonstrated in a murine model (8). Human polyclonal antibodies cross-react in a similar manner, although the nature of this cross-reactivity is still unclear (8). There is also a correlation between immune responsiveness and the HLA-haplotype. Whereas, HLA-B35 might be associated with pustular skin diseases, HLA-A2 is a risk factor for the development of rheumatoid arthritis (9). Pharyngeal infection with group A-β hemolytic streptococcus may lead to acute rheumatic fever in individuals who have an inherited recessive gene closely linked to the HLA locus that is responsible for high responsiveness to the streptococcal polysaccharide antigen of the cell wall (10). The production of bacterial products (skin toxins) has also been proposed (11). In the present case, we were unable to completely exclude the possibility that PAG was caused by an allergic preservative reaction.

Patients with PAG are susceptible to mucosal involvement, glomerulonephritis, and arthralgias. The clinical manifestations are similar to AGEP and GPP. The main similarity of the three disease processes in the differential diagnosis is the association of PAG with streptococcal infection. Prolonged pharyngeal infections and short-term antibiotic therapy (< 10 days) increases the likelihood of subsequent rheumatic fever, especially in HLA-A2- and HLA-B35-positive patients. Our patient was HLA-A2- and HLA-B35-negative, thus no complications, such as rheumatic fever, occurred during a 1-year follow-up. Therefore, it is essential to make the distinction between PAG, AGEP, and GPP so that appropriate therapy can be administered.

The current case report highlights the need to consider the possibility of PAG when pustules appear on the skin and a culture from a throat swab is positive for group A β-hemolytic streptococcus. HLA-A2 and HLA-B35 status should be determined for timely diagnosis and thorough treatment, otherwise the risk of rheumatic fever is increased.

## Data availability statement

The original contributions presented in the study are included in the article/supplementary material, further inquiries can be directed to the corresponding author.

## Ethics statement

Written informed consent was obtained from the individual(s) for the publication of any potentially identifiable images or data included in this article. Written informed consent was obtained from the participant/patient(s) for the publication of this case report.

## Author contributions

Assessment of study patients: XL, YZ, and HC. Drafting of the manuscript: XL, HB, and LR. Critical revision of the manuscript: HC, SG, and YD. Anatomopathological analysis: HL. All authors contributed to the article and approved the submitted version.
